# Infrared optical imaging of matrix metalloproteinases (MMPs) up regulation following ischemia reperfusion is ameliorated by hypothermia

**DOI:** 10.1186/1471-2202-13-76

**Published:** 2012-06-28

**Authors:** Philip A Barber, David Rushforth, Smriti Agrawal, Ursula I Tuor

**Affiliations:** 1 Department of Clinical Neurosciences, Experimental Imaging Centre and Hotchkiss Brain Institute, Faculty of Medicine, University of Calgary, Calgary, Canada; 2 IBD West, National Research Council,, , Canada; 3 Experimental Imaging Centre, B153, 3330 Hospital Dr NW, University of Calgary, Calgary, AB, T2N 2T9, Canada

## Abstract

**Background:**

We investigated the use of a new MMP activatable probe MMPSense^™^ 750 FAST (MMPSense750) for *in-vivo* visualization of *early* MMP activity in ischemic stroke. Following middle cerebral artery occlusion (MCAO) optical imaging was performed. Near-infrared (NIR) fluorescent images of MMPSense activation were acquired using an Olympus fluorescent microscope, 1.25x objective, a CCD camera and an appropriate filter cube for detecting the activated probe with peak excitation and emission at 749 and 775 nm, respectively. Images were acquired starting at 2 or 24 hours after reperfusion over the ipsilateral and contralateral cortex before and for 3 hours after, MMPSense750 was injected.

**Results:**

Increased intensities ipsilaterally were observed following MMPSense750 injection with ischemic injury but not in sham animals. There were significant ipsilateral and contralateral differences at 15 minutes (P <0.05) in early ischemic reperfusion and at time 0 in 24 hours post ischemia (P <0.05) which persisted at 180 minutes in both these groups (P <0.01), but not following sham surgery. The increase in ipsilateral signal intensity was attenuated by hypothermia. These observations corresponded with a significant increase in the total MMP-9 protein levels, 5 and 24 hours following ischemia reperfusion (P <0.05) and their reduction by hypothermia.

**Conclusions:**

Matrix-metalloproteinase upregulation in ischemia reperfusion can be imaged acutely *in-vivo* with NIRF using MMPSense750. Hypothermia attenuated both the optical increase in intensity after MMPSense750 and the increase in MMP-9 protein expression supporting the proof of concept that NIRF imaging using MMPSense can be used to assess potential therapeutic strategies for stroke treatment.

## Background

Stroke is the third leading cause of death and the leading cause of long-term disability in adults. Several members of the matrix metalloproteinase (MMPs) family have been implicated to have detrimental roles in stroke [1,2]. Specifically, the gelatinases MMP-2 and MMP-9 have previously been considered to specifically injure the important components of the basal lamina around the cerebral blood vessels that precede microvascular damage in cerebral ischemia [1]. This leads to disruption of the blood brain barrier, edema, and hemorrhagic transformation in animal models of ischemia [3,4]. Once within the CNS, MMPs continue to damage CNS tissue. In general higher MMP-9 levels are shown to correlate significantly with larger infarct volume, severity of stroke, reduced survival of neurons and worse functional outcome [5], and therefore, seem an appropriate target for a robust neuroprotectant such as hypothermia [6,7].

Most current studies investigating cerebral ischemia and stroke detect MMPs *in vitro* using techniques such as Western Blotting [8], ELISA [9] RT-PCR or staining with anti-MMP antibodies [10]. While these are good techniques they fall short when reporting accurate levels of the active form of MMP, are limited in their sensitivities of MMP detection, and may not be a precise depiction of MMP activity *in vivo*. *In vivo* visualization of MMP activity would provide important information regarding the spatial and temporal expression of MMP enzymatic activity with respect to the pathophysiology of the disease and to monitor response to interventions, such as therapeutic hypothermia.

NIRF emitting probes offer the advantage of increased depth of detection. Recently probes that fluoresce upon proteolytic cleavage by MMP’s have become available with the potential advantage of exhibiting low background fluorescence. One such probe, MMPSense680 (Perkin Elmer Inc (previously VisEn Medical), Boston, MA USA) with a peak excitation at approx 680 nm and emission at 700 nm, was shown to produce increased fluorescent signal considered related to MMP mediated cleavage in diseases including stroke [11,12]. Activation of the probe is reported to occur by a broad range of MMP’s including MMP 2, 3, 9, and 13 [11]and the manufacturer recommends imaging at 24 hr following administration.

The objective of the present study was to determine whether an alternate MMP activatable probe with a shorter optical imaging time, MMPSense^™^ 750 FAST (MMPSense750), could be used for visualization of MMP activity in the *early* stages of ischemia reperfusion in a mouse model of stroke. This probe also has fluorescent properties in the near infrared range (peak excitation and emission at approx 749 nm and 775 nm, respectively) and according to the manufacturer is sensitive to cleavage and optical activation by various MMP’s including MMP2,3,7,9,12 and MMP13. In order to investigate the responsiveness of the probe to alterations in MMP activity, the current study also examined whether the MMPSense750 probe would detect reductions in MMP’s associated with a therapeutic intervention following stroke We chose mild hypothermia for its well documented therapeutic reductions in various MMPs in a number of neurological diseases including stroke [13-16]. In parallel with *in vivo* studies, characteristics of the probe were investigated *in vitro*.

## Methods

### Optical activation of MMPSense750 *in vitro*

To test the optical changes in MMPSense750 (Elmer Perkin Inc, Boston MA, USA) *in vitro*, we investigated fluorescent intensity changes following its addition to several different test solutions. The test solutions consisted of blood (from an animal 2 hours following MCAO plus reperfusion), phosphate buffer (PBS), and PBS with an enzyme (tryspin, 1 mg/ml) that would cleave and activate the optical probe. The test solution (95ul) was added to a 96 well plate and a zero reading was taken. Then 5ul of MMPSense750, was added to the wells and shaken. Near-Infrared Images were acquired repeatedly over 30 minutes from which intensity changes were measured. In control experiments, PBS was added instead of MMPSense750. Experiments were repeated in duplicate or triplicate. .

### Near infrared fluorescent imaging

Near-Infrared images were acquired using a 1.25× objective (PLAN-APO) on an Olympus BX51 fluorescent microscope fitted with a back thinned CCD camera (Q-imaging Rolera) and an Olympus Cy7-B-OMF-ZERO filter cube with excitation/emission bands of 665-750 nm and 765 to 855 nm, respectively. Digital images (14 bit monochromatic) were acquired with a temporal resolution of 4 sec and a pixel size of 12.6 μmx12.6 μm (512 × 512 pixels). Emission intensity was measured as mean gray levels in regions of interest using Image-J software (Research Services Branch, National Institute of Mental Health, Bethesda, Maryland, USA).

### *In vivo* experiments

Male C57Bl/6 mice (3 months old, 25 to 35 g; Charles River Breeding Laboratories, Ontario, Canada) were prepared for transient MCAO using the intraluminal filament method [17]. A total of forty-six animals were included in the study. Of these 25 were dedicated for optical imaging and 21 for quantification of MMP protein expression with ELISA. The animal groups were SHAM (N = 13), early ischemia reperfusion (N = 14), early ischemia reperfusion with the induction of hypothermia (33 °C) (N = 8), and ischemia reperfusion at 24 hours (N = 11). All experiments and procedures were approved by the local University of Calgary and National Research Council animal care committees and were in accordance with the Canadian Council of Animal Care guidelines.

### Transient focal ischemia

Anesthesia was induced with isoflurane (3% initial, 1% to 1.5% maintenance) in O_2_ and air (80%:20%). Briefly, under the operating microscope, the left common carotid artery (CCA), the left external carotid artery (ECA), and the left internal carotid artery (ICA) were isolated and a 6–0 suture was tied at the origin of the ECA and at the distal end of the ECA. The left CCA and ICA were temporarily occluded. The silicon-coated nylon suture (diameter 180-220 μm) was introduced into the ECA and inserted into the ICA approximately 9 to 10 mm from the carotid bifurcation until meeting resistance and effectively blocking the middle cerebral artery. The suture remained inserted for 30 minutes, after which it was removed and the ECA was permanently tied.

### Measurement of cerebral blood flow

Transcranial measurements of cerebral blood flow (CBF) were made by laser-Doppler flowmetry (LDF) while the animal was under general anesthesia (Perisoft Version 1.3; Perimed Inc). A 0.5-mm diameter microfiber laser-Doppler probe (Probe 418; Perimed) was attached to the skull with cyanoacrylate glue 6 mm lateral and 1 mm posterior of bregma. Data were expressed as a mean percentage of the baseline pre-ischemia value. The occlusion was considered adequate if ≥70% reduction in cortical CBF occurred immediately after placement of the intraluminal occluding suture; otherwise, mice were excluded.

### Temperature regulation

Mice were implanted with intra-abdominal radiofrequency probes (TA10TA-F20; Transoma Medical) 7 days before MCA occlusion. Core temperature was sampled every 30 seconds using receivers Activity and temperature data was collected every 30 seconds during movement of the animal over the receiver (RLA-1020; Data Sciences Int.) interfaced to a computer running ART 2.2. During the surgical procedure, the animals were regulated at 36.5 °C (total time 2 hours) by a 125-W heating lamp.

### Intravital fluorescence measurements

Ninety minutes or 24 hours post-reperfusion, a lateral tail vein was cannulated for intravenous infusion of MMPSense750. Core body temperature was continuously monitored and maintained using a rectal probe with feedback to a heating lamp for the duration of the experiment at 36.5 °C for normothermia or 33.5 °C for hypothermia (started at the onset of reperfusion and maintained for the duration of the experiment). Because initial experiments had difficulty imaging MMPSense 750 through the skull we created a window using a high-speed micro-drill and saline cooling to expose the left parietal bone between the lambdoidal suture, sagittal suture and coronal suture. A control window was made on the contralateral side.

Animals were placed on a custom designed heated platform attached to the stage of the Olympus BX51 microscope. One hundred and twenty minutes after reperfusion, animals were imaged over ipsilateral and contralateral parietal cortex to obtain baseline background measures and then 150 μl MMPSense750 was infused over 60 seconds. Optical imaging data was then acquired every 5–10 minutes over 3 hours. At 3 hours animals were euthanized and the entire brain was removed and imaged *ex-vivo* using a FITC filter cube (maximal excitation and emission of 490 and 520 nm, respectively) instead of the NIR filter cube. Endogenous blue/green fluorescence was increased in the ischemic hemisphere and this fluorescence was considered related to increased levels of mitochondrial coenzymes (e.g. NADH and NADPH) [18] produced within the ischemic lesion[19] thereby providing an estimate of the extent of the stroke. Brains were dissected and prepared for histology or ELISA assay of MMP-9 and 2.

### ELISA assay

Brains were cut into thick axial sections in PBS at 4 °C. Stroke lesion was identified by the endogenous region of autofluorescence with FITC imaging and a sample was dissected along with an equivalent sample in the contralateral region. Tissue was homogenized in radioimmunoprecipitation assay (RIPA) buffer with Ethylenediaminetetraacetic acid (EDTA)-free protease inhibitors (Roche) by sonication for 2 minutes on ice. Homogenates were centrifuged (1000 G, 5 mins) and supernatants collected for analysis. ELISA kits were used according to the manufacturer’s instructions for measurement of murine total and pro- murine MMP-9, (Quantikine ELISA Kits,R & D systems) and murine MMP-2 (Abnova). Supernatants were assayed for protein concentration (Pierce, BCA assay kit) and results were expressed as ng MMP per mg total protein.

### Histology

Animals were euthanized with sodium pentobarbital (70 mg/kg intraperitoneally) and transcardially perfused with 0.9% saline, followed by 4% paraformaldehyde. The brain was then embedded in paraffin and sections were cut at 6 μm thickness and stained with hematoxylin and eosin. The distance between sections was ≈ 1 mm. Paraffin sections were de-waxed in xylene and rehydrated. Antigen retrieval was performed by boiling sections in citrate buffer for 2 minutes. Sections were then washed in PBS (0.01 M),Triton X100(1%) for 10 minutes followed by 2 subsequent washes in PBS(0.01 M) for 5mins. Normal goat serum (10%)/PBS(0.01 M) was then applied to the sections for 30 minutes at room temperature to block non-specific binding. Primary antibody (MAB305, Millipore) Rabbit anti MMP-9 n-terminal was diluted 1/200 in PBS (0.01 M)/BSA 1% and applied to sections and then incubated at 4 °C overnight. Sections were washed 3 times in PBS (0.01 M) for 5mins. Goat anti-rabbit TRITC secondary antibody diluted 1/1000 in PBS was then applied to the sections and incubated for 2 hours at room temperature. Sections were then washed at 3 x 10 minutes in PBS (0.01 M) and mounted with DAKO hardset fluorescent mounting media. Positive fluorescent staining was captured using an Olympus BX61 microscope (objective *x*2) and Microfire Optronics digital camera.

### Statistics

The data are presented as mean ± S.D., and were analyzed using a One way repeated measures Analysis of Variance (ANOVA), non parametric Dunnet post test and Kruskal Wallis ANOVA for Ranks for left right comparison of continuous data. Differences were considered significant at p <0.05.

## Results

### MMPSense750 has a significant baseline signal and is unaffected by blood from stroke animals examined *in vitro*

**Figure 1:**
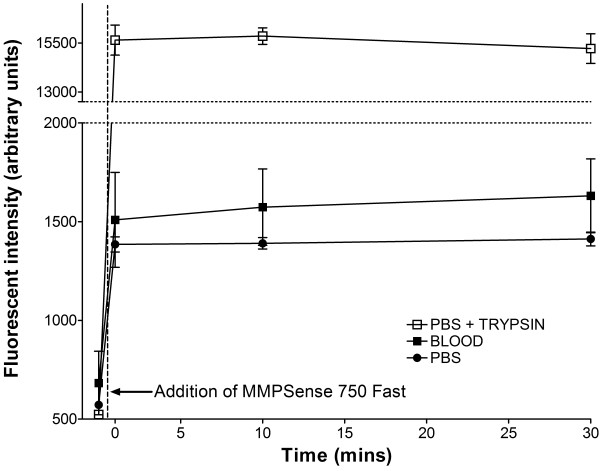
**Effect of MMPSense750 cleavage*in vitro.*** Addition of 5 μl MMPSense750 to 95 μl blood from animals following stroke produced no significant additional increase in intensity when compared to its addition to a control solution of phosphate buffer-(PBS). In contrast, the addition of MMPSense750 to a solution of 45 μl PBS + 50 μl trypsin resulted in a marked increase in fluorescent intensity (P <0.001, 2 way ANOVA).

The addition of MMPSense750 to a sample of PBS resulted in an immediate increase in the NIR fluorescent intensity detected indicating that the optical solution contains unconjugated probe that is optically active and provides a background level of grey scale fluorescence intensity (Figure 1). The addition of MMPSense750 to blood from stroke animals did not result in a substantial additional increase in gray scale intensity relative to PBS suggesting that there is little activation of the probe following the administration of blood (Figure 1). The addition of 5 μl PBS instead of MMPSense750 to blood did not result in a significant change in intensity (data not shown). However, the addition of MMPSense750 to 45 μl PBS + 50 μl trypsin resulted in a marked increase in fluorescent signal indicative of optical activation of the probe by proteolytic cleavage with trypsin.

### MMPSense750 is activated in early ischemia reperfusion

The cortical CBF change in all animals successfully reached the set threshold (>70% decrease from baseline). The mean LDF reduction for each group was: early ischemia reperfusion 2HR (84%,SD 3.6), early ischemia reperfusion with the induction of hypothermia (33°C) (81%, SD2.8), and ischemia reperfusion at 24 hours (93%,SD5.9). There was no statistical difference between animal groups (P = 0.33).

**Figure 2:**
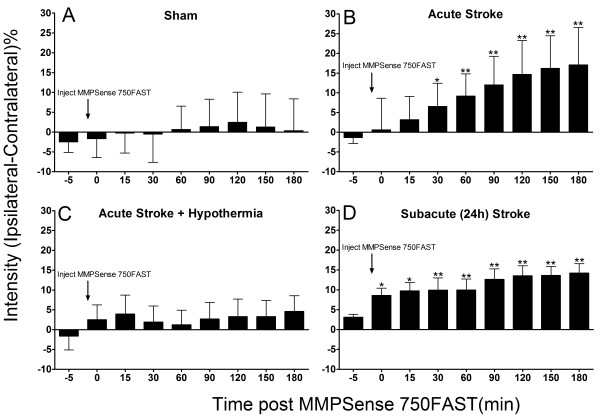
**MMPSense750 activation at acute (2 hour) and subacute (24 hour) time points after reperfusion from MCAO.** MMPSense750 injection in sham animals (**A**) resulted in no significant ipsilateral versus contralateral differences in fluorescent intensity whereas with acute MCAO reperfusion (**B**), there were significant differences in fluorescent intensity by 30 minutes (P <0.05) post injection that persisted until 180 minutes. At 24 hours post ischemia there were immediate ipsilateral-contralateral differences following probe injection that persisted for all the times examined. The increase in ipsilateral signal intensity was attenuated by hypothermia (**D**). * P <0.05, ** p <0.01 comparison to pre-injection value (One way repeated measures ANOVA, non parametric Dunnet post test).

Two hours after the onset of reperfusion post ischemia, administration of MMPSense750 resulted in an increase of NIR fluorescent intensity (Figure 2.B P <0.05) levels at 30–180 minutes following injection not seen in sham controls (Figure 2A). Acute stroke combined with hypothermia reduced MMPSense750NIR intensity levels (Figure 2C). The optical intensity increase persisted up to the final recording at 5 hours of ischemia reperfusion. Similar results were observed following injection of MMPSense750 at 24 hour stroke, the difference being that the initial rate of change and maximal optical intensity occurred earlier within minutes of administering the MMPSense 750 (Figure 2D) indicative of the presence of substantial quantities of MMPs/proteases in the cortex resulting in an abrupt activation of the MMPSense750 rather than a steady rise in MMPs or intensity at the acute time point (Figure 2 B). No statistical difference in optical intensity between the left and right hemisphere was seen in sham operated animals or in animals with hypothermia induced immediately after 30 minutes of MCAO (Figure 2 A,C).

### MMP-9 protein is predominately upregulated in early ischemia reperfusion and at 24 hours

**Figure 3:**
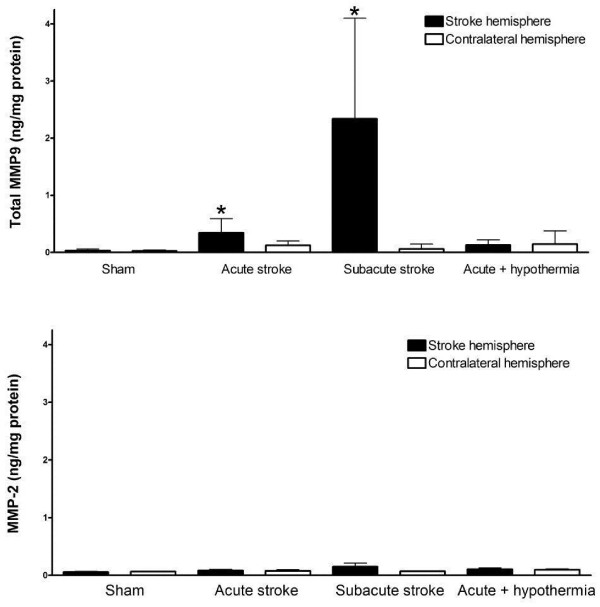
**MMP 9 and MMP2 expression following early (5hour) and subacute (24 hour) ischemia reperfusion.** An increased level of MMP expression was apparent at 5 hours and 24 hours post ischemia reperfusion (*P <0.05; different from corresponding Sham surgery control; Kruskal Wallis ANOVA and Dunn’s test). An increase in MMP 2 was not observed either in early (5 hr) or subacutely (24 hour) ischemic reperfusion.

**Figure 4:**
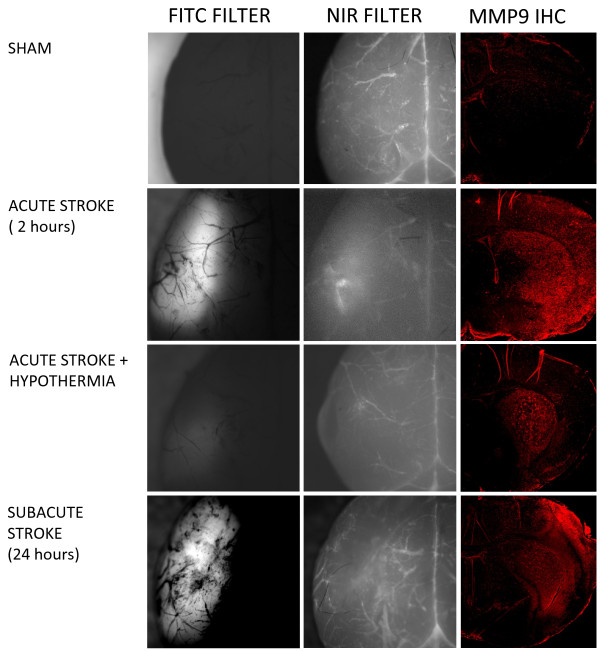
**Fluorescent images of representative brains following injection with MMPSense750.** Autofluorescence seen in the FITC channel (left hand column) is considered to indicate increased coenzyme levels such as NADH revealing the location of the stroke. A localized increase in intensity using the NIR channel (mid column) is observed following injection of MMPSense750, both acutely and subacutely following stroke but less appreciably in sham and hypothermia treated animals. These near infrared intensity changes correspond to MMP9 increases in immunohistochemical staining of coronal frozen sections shown from representative animals (right hand column).

Quantification of total MMP-9 protein from brain homogenates using ELISA revealed a predominant increase in MMP-9 levels at 5 and 24 hours post ischemia reperfusion (Figure 3), with higher MMP-9 levels detected in the stroke hemisphere during a subacute stroke versus and acute stroke (Figure 3). This increase in MMP-9 levels in acute stroke were found to be attenuated by hypothermia (P <0.05) (Figure 3). Changes in MMP-2 protein expression were not detected at either time point (Figure 3). This increase in MMP-9 was confirmed with positive immunofluoresence staining for MMP-9 within the ipsilateral stroke hemisphere and corresponding reductions of such staining with hypothermia (Figure 4). Changes corresponding to these altered MMP-9 levels were visualized *in vivo* using NIR fluorescence imaging of the activated MMPSense750 in the stroke region (Figure 4) compared to sham controls. In animals maintained hypothermic for 5 hours following stroke, there was an attenuation of the activated fluorescent signal intensity (Figure 4).

## Discussion

This study reports a novel early up-regulation of MMP-9 in ischemic reperfusion. The acute *in-vivo* detection of MMP upregulation following ischemic stroke has not been reported previously. Importantly, we describe an innovative technique by which the increase in MMP-9 levels can be imaged *in-situ* within the first few hours of ischemia reperfusion using NIR fluorescent imaging and an activatable optical probe MMPSense750. Furthermore, we demonstrate that hypothermia attenuates the optical intensity increase in MMPSense750 and the increase in MMP-9 protein expression among the broad range of MMP’s potentially detected by MMPSense750, (e.g. MMP 2, 3, 7, 9, 12, and 13). Our data provides evidence that MMPSense750 can detect activity of MMPs *in-vivo* and taken together our findings support the proof of concept that NIR fluorescent imaging can be used *in situ* to assess the mechanisms involved in cerebral ischemia.

The expression of MMP-9 corresponded well with the spatial and temporal activation of MMPSense750. The time dependent activities of MMPs have been previously well documented [20,21]. Of the two MMPs - 9 and 2 that are consistently reported to be up-regulated in ischemic stroke, we found MMP-9 to be the most strongly expressed in early ischemia reperfusion and later at 24 hours post reperfusion. The increased activity of the MMPSense750 sense probe was evident regionally and temporally in both the infarct and peri-infarct regions when compared with the regions of endogenous FITC fluorescence considered to represent areas of predominantly increased NADH within the ischemic lesion [18,19]. The early increase in NIR optical intensity would suggest that MMPSense750 is being activated by increased MMP activity either at the intraluminal surface by activated leukocytes or at the basal lamina [16]. It is important to note that when using this probe it is necessary to control for presence of unconjugated probe and potential systemic activation of the probe *in vivo*, because when we added MMPSense750 to PBS fluorescent intensity immediately increased and MMPSense750 administered to sham operated mice produced immediate low level near infrared fluorescence intensity increases equally distributed over both hemispheres.

Non invasive imaging of MMP activity remains a clinically important unmet need. In a recent exploratory study, a NIR fluorescent probe with excitation emission in the 680 nm wavelength range was used to detect MMP activity in an animal model of stroke using NIRF imaging accompanied by magnetic resonance and radioisotope imaging techniques [12]. In this study, the utility of this 680 nm probe was investigated only at 24 hours after ischemia. For optimal clinical application, it is important to have an activatable probe that can also detect early MMP upregulation following cerebral ischemia and the present results demonstrate MMPSense750 can detect earlier acute changes in MMP/protease activity.

There were recognized limitations of our study. We used gelatin zymography to detect MMP-2 and 9 in brain tissues, and the levels of these MMPs were too low to be detected by this technique suggesting the importance of our imaging method, which is sensitive enough to detect low levels of MMP activity. The fact that we were not able to detect large quantities of pro-MMP-9 would suggest that the optical changes measured included activated MMP-9. Clearly also possible is that other MMP’s contributed to the MMPSense intensity increases observed. Furthermore, our observation that trypsin activates MMPSense750 *in vitro* supports the conclusion that this probes activation can be induced by other proteases such as serine proteases in addition to MMPs. Another limitation was our inability to readily image NIR fluorescent changes though the skull. Non invasive detection is theoretically possible with NIR probes but considering the small magnitude of the changes observed we believe this would need substantial technical improvements with advances in non-invasive imaging systems and probe sensitivity. In addition to the small changes in signal intensity, the need for background correction for this particular probe may limit its clinical applicability.

A novel component of this study was the *in vivo* demonstration that moderate hypothermia induced after ischemia reperfusion reduced both the NIR optical signal intensity and also the level of MMP-9 protein detected. Previous studies from MMP gene knockout mice and those using MMP pharmacological inhibitors suggest that the MMPs may be attractive therapeutic targets for stroke [22-24]. It has been demonstrated that moderate hypothermia (32-34°C) protects the basal lamina, reduces infarct volume and hemorrhage, and reduces MMP-9 [25,26]. However, emerging data now also suggests that some aspects of MMP activity during the delay of neuroinflammatory response may contribute to re-modeling in stroke recovery [27]. Therefore, defining the time dependent relationship of MMP activation has been increasingly emphasized and timing of therapeutic strategies require detailed refinement to avoid the potential theoretical deleterious effects of MMP inhibition on stroke remodeling. In this capacity the use of experimental tools such as near NIR fluorescent probes and NIR imaging may become invaluable both in pre clinical and clinical testing.

## Conclusions

This study shows that matrix-metalloproteinase upregulation in ischemia reperfusion can be imaged acutely *in-vivo* with NIRF using MMPSense750. Hypothermia attenuated both the optical increase in intensity after MMPSense750 and the increase in MMP-9 protein expression supporting the proof of concept that NIRF imaging using MMPSense can be used to assess potential therapeutic strategies for stroke treatment.

## Competing Interests

The author(s) declare that they have no competing interests.

## Authors’ Contributions

PB conceptualized the study design, was involved in data analysis, coordinated all aspects of the study, wrote the manuscript and critically appraised the manuscript. DR performed the *in vivo* stroke animal studies and contributed to data acquisition and analysis. SA was involved in data acquisition and tissue processing as well as the critical appraisal of the manuscript. UT was involved in the conceptualization of the study, data analysis and acquisition, in addition to contributing to critical appraisal and writing of the manuscript. All authors read and approved the final manuscript.

## References

[B1] Del Zoppo GJ The neurovascular unit, matrix proteases, and innate inflammation Ann N Y Acad Sci 2010 1207 46 49 10.1111/j.1749-6632.2010.05760.x 20955425 PMC4547552

[B2] Yong VW Krekoski CA Forsyth PA Bell R Edwards DR Matrix metalloproteinases and diseases of the CNS Trends Neurosci 1998 21 75 80 10.1016/S0166-2236(97)01169-7 9498303

[B3] Rosenberg GA Navratil M Barone F Feuerstein G Proteolytic cascade enzymes increase in focal cerebral ischemia in rat J Cereb Blood Flow Metab 1996 16 360 366 8621740 10.1097/00004647-199605000-00002

[B4] Sumii T Lo EH Involvement of matrix metalloproteinase in thrombolysis-associated hemorrhagic transformation after embolic focal ischemia in rats Stroke 2002 33 831 836 10.1161/hs0302.104542 11872911

[B5] Ramos-Fernandez M Bellolio MF Stead LG Matrix metalloproteinase-9 as a marker for acute ischemic stroke: a systematic review J Stroke Cerebrovasc Dis 2011 20 47 54 10.1016/j.jstrokecerebrovasdis.2009.10.008 21044610

[B6] MacLellan CL Clark DL Silasi G Colbourne F Use of prolonged hypothermia to treat ischemic and hemorrhagic stroke J Neurotrauma 2009 26 313 323 10.1089/neu.2008.0580 19216634

[B7] Choi HA Badjatia N Mayer SA Hypothermia for acute brain injury-mechanisms and practical aspects Nat Rev Neurol 2012 8 4 214 22 22371279 10.1038/nrneurol.2012.21

[B8] Pires PW Rogers CT McClain JL Garver HS Fink GD Dorrance AM Doxycycline, a matrix metalloprotease inhibitor, reduces vascular remodeling and damage after cerebral ischemia in stroke-prone spontaneously hypertensive rats Am J Physiol Heart Circ Physiol 2011 301 1 H87 97 10.1152/ajpheart.01206.2010 21551278 PMC3129920

[B9] Rodriguez-Gonzalez R Sobrino T Rodriguez-Yanez M Millan M Brea D Miranda E Association between neuroserpin and molecular markers of brain damage in patients with acute ischemic stroke J Transl Med 2011 9 58 10.1186/1479-5876-9-58 21569344 PMC3113955

[B10] Chaudhry K Rogers R Guo M Lai Q Goel G Liebelt B Matrix metalloproteinase-9 (MMP-9) expression and extracellular signal-regulated kinase 1 and 2 (ERK1/2) activation in exercise-reduced neuronal apoptosis after stroke Neurosci Lett 2010 474 109 114 10.1016/j.neulet.2010.03.020 20298757

[B11] Yoon SM Myung SJ Ye BD Kim IW Lee NG Ryu YM Near-infrared fluorescence imaging using a protease-specific probe for the detection of colon tumors Gut Liver 2010 4 488 497 10.5009/gnl.2010.4.4.488 21253297 PMC3021604

[B12] Klohs J Baeva N Steinbrink J Bourayou R Boettcher C Royl G In vivo near-infrared fluorescence imaging of matrix metalloproteinase activity after cerebral ischemia J Cereb Blood Flow Metab 2009 29 1284 1292 10.1038/jcbfm.2009.51 19417756

[B13] Truettner JS Alonso OF Dalton DW Influence of therapeutic hypothermia on matrix metalloproteinase activity after traumatic brain injury in rats J Cereb Blood Flow Metab 2005 25 1505 1516 10.1038/sj.jcbfm.9600150 15959464

[B14] Baumann E Preston E Slinn J Stanimirovic D Post-ischemic hypothermia attenuates loss of the vascular basement membrane proteins, agrin and SPARC, and the blood–brain barrier disruption after global cerebral ischemia Brain Res 2009 1269 185 197 19285050 10.1016/j.brainres.2009.02.062

[B15] Horstmann S Koziol JA Martinez-Torres F Nagel S Gardner H Wagner S Sonographic monitoring of mass effect in stroke patients treated with hypothermia. Correlation with intracranial pressure and matrix metalloproteinase 2 and 9 expression J Neurol Sci 2009 276 75 78 10.1016/j.jns.2008.08.038 18834996

[B16] Nagel S Su Y Horstmann S Heiland S Gardner H Koziol J Minocycline and hypothermia for reperfusion injury after focal cerebral ischemia in the rat: effects on BBB breakdown and MMP expression in the acute and subacute phase Brain Res 2008 1188 198 206 18031717 10.1016/j.brainres.2007.10.052

[B17] Barber PA Hoyte L Colbourne F Buchan A Temperature regulated model of focal ischemia in the mouse: a study with histopathological and behavioural outcomes Stroke 2004 35 1720 1725 10.1161/01.STR.0000129653.22241.d7 15155973

[B18] Kasischke KA Lambert EM Panepento B Sun A Gelbard HA Burgess RW Two-photon NADH imaging exposes boundaries of oxygen diffusion in cortical vascular supply regions J Cereb Blood Flow Metab 2011 31 68 81 10.1038/jcbfm.2010.158 20859293 PMC3049466

[B19] Mayevsky A Rogatsky GG Mitochondrial function in vivo evaluated by NADH fluorescence: from animal models to human studies Am J Physiol Cell Physiol 2007 292 C615 C640 16943239 10.1152/ajpcell.00249.2006

[B20] Chang DI Hosomi N Lucero J Heo JH Abumiya T Mazar AP Activation systems for latent matrix metalloproteinase-2 are upregulated immediately after focal cerebral ischemia J Cereb Blood Flow Metab 2003 23 1408 1419 14663336 10.1097/01.WCB.0000091765.61714.30

[B21] Yang Y Estrada EY Thompson JF Liu W Rosenberg GA Matrix metalloproteinase-mediated disruption of tight junction proteins in cerebral vessels is reversed by synthetic matrix metalloproteinase inhibitor in focal ischemia in rat J Cereb Blood Flow Metab 2007 27 697 709 16850029 10.1038/sj.jcbfm.9600375

[B22] Dong X Song YN Liu WG Guo XL Mmp-9, a potential target for cerebral ischemic treatment Curr Neuropharmacol 2009 7 269 275 10.2174/157015909790031157 20514206 PMC2811860

[B23] Ponnampalam SN Mayberg MR Mediators of blood–brain barrier disruption and potential therapeutic interventions for protection of the barrier following focal ischemia Clin Neurosurg 2004 51 112 119 15571135

[B24] Horstmann S Kalb P Koziol J Gardner H Wagner S Profiles of matrix metalloproteinases, their inhibitors, and laminin in stroke patients: influence of different therapies Stroke 2003 34 2165 2170 10.1161/01.STR.0000088062.86084.F2 12907822

[B25] Burk J Burggraf D Vosko M Dichgans M Hamann GF Protection of cerebral microvasculature after moderate hypothermia following experimental focal cerebral ischemia in mice Brain Res 2008 1226 248 255 18586014 10.1016/j.brainres.2008.06.015

[B26] Lee JE Yoon YJ Moseley ME Yenari MA Reduction in levels of matrix metalloproteinases and increased expression of tissue inhibitor of metalloproteinase-2 in response to mild hypothermia therapy in experimental stroke J Neurosurg 2005 103 289 297 10.3171/jns.2005.103.2.0289 16175859

[B27] Lo EH A new penumbra: transitioning from injury into repair after stroke Nat Med 2008 14 497 500 10.1038/nm1735 18463660

